# Fan-out in gene regulatory networks

**DOI:** 10.1186/1754-1611-4-16

**Published:** 2010-12-17

**Authors:** Kyung H Kim, Herbert M Sauro

**Affiliations:** 1 Department of Bioengineering, University of Washington, William H. Foege Building, Box 355061, Seattle, WA 98195-5061, USA

## Abstract

**Background:**

In synthetic biology, gene regulatory circuits are often constructed by combining smaller circuit components. Connections between components are achieved by transcription factors acting on promoters. If the individual components behave as true modules and certain module interface conditions are satisfied, the function of the composite circuits can in principle be predicted.

**Results:**

In this paper, we investigate one of the interface conditions: fan-out. We quantify the fan-out, a concept widely used in electrical engineering, to indicate the maximum number of the downstream inputs that an upstream output transcription factor can regulate. The fan-out is shown to be closely related to retroactivity studied by Del Vecchio, et al. An efficient operational method for measuring the fan-out is proposed and shown to be applied to various types of module interfaces. The fan-out is also shown to be enhanced by self-inhibitory regulation on the output. The potential role of an inhibitory regulation is discussed.

**Conclusions:**

The proposed estimation method for fan-out not only provides an experimentally efficient way for quantifying the level of modularity in gene regulatory circuits but also helps characterize and design module interfaces, enabling the modular construction of gene circuits.

## Background

Engineering relies on modular composition, that is, the ability to combine functional units with the knowledge that the intrinsic properties of each module is unaffected to a large degree by the composition. In biology, the notion of a modular component is less clear, or at least biology has multiple definitions depending on context [1]. Here a module is defined as a self-contained functional unit whose intrinsic properties are independent of the surrounding milieu. This definition is similar to that used in engineering. For example, the intrinsic properties of a CMOS (complementary metal oxide semiconductor) NAND gate [2] is unaffected (within certain design constraints) when connected to other CMOS logic gates. That is, a NAND gate remains a NAND gate no matter what it is connected to. This property allows engineers to design, predict, and fabricate complex circuits at very low cost. The question whether such self-contained and functionally independent modules exist at the biological cellular network level is still an ongoing research problem [3]. In this paper, the design of modular synthetic components [4-10] is considered, and the question of modularity in natural complex systems is avoided.

In the most abstract sense, a module can be defined as follows. Given a functional unit *M *with input *I *and output *O*, a relation between the input and output can be defined as *O *= *M*(*I*). Given two functional units, *M*_1 _and *M*_2_, where the output of *M*_1 _serves as the input to *M*_2_, then *M*_1 _and *M*_2 _are defined as modules if the relation, *O*_2 _= *M*_2_(*M*_1_(*I*_1_)) is true. This simply means that in connecting *M*_1 _and *M*_2 _together, *M*_2 _has no effect on the functional characteristics of *M*_1 _and vice versa.

**Figure 1:**
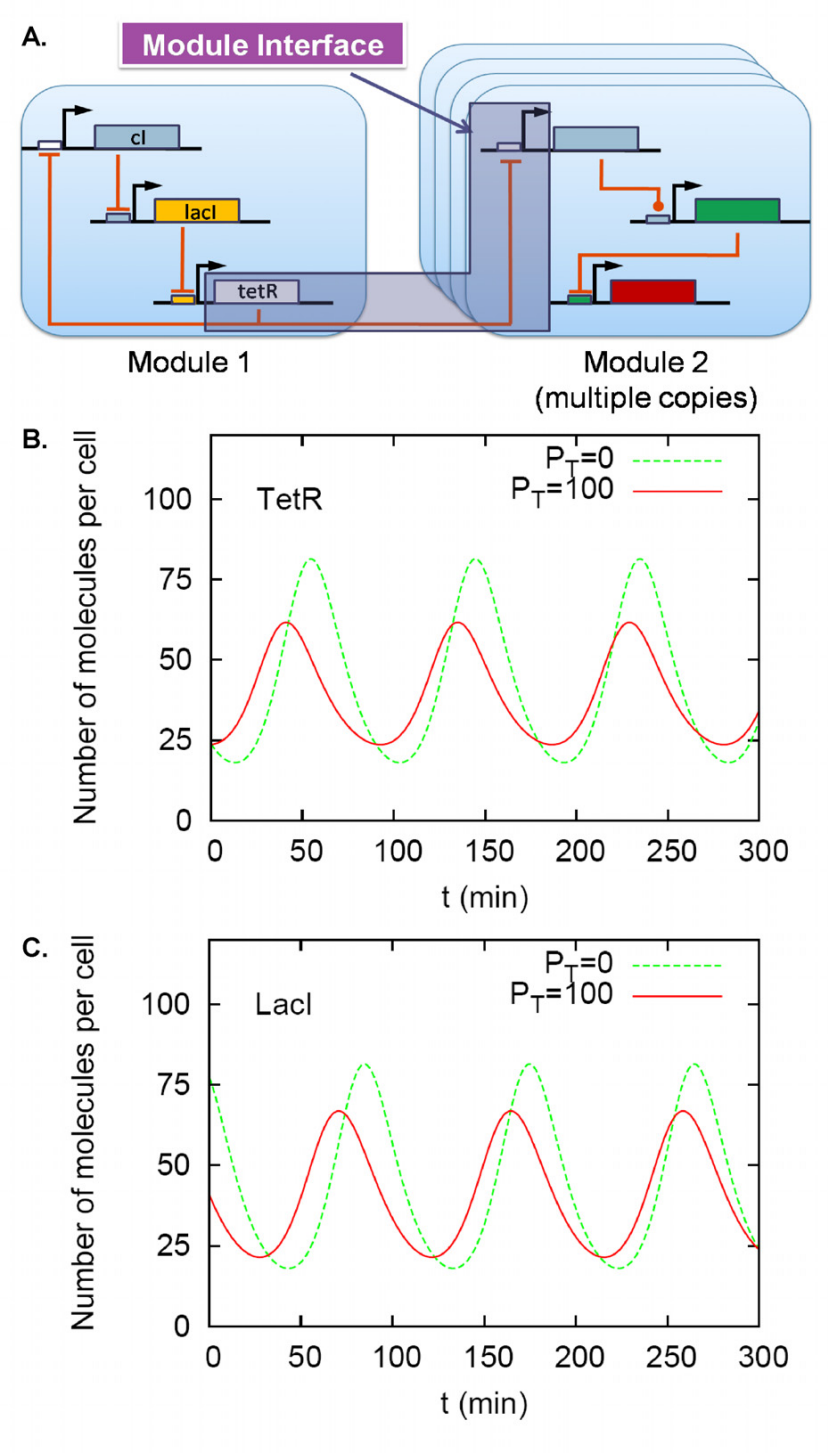
**Gene circuit modules and their interface process**. (A) The repressilator (Module 1) [30] regulates multiple copies of a downstream module (Module 2). The multiple copies can be realized by placing the downstream module in a plasmid. TetR repressors (output of Module 1) regulate their specific downstream-module promoters (input of Module 2). A module interface process (MIP) defines the collection of the processes of *tetR*-transcription and translation and its specific downstream-module regulation. As the number of the downstream-module promoter (*P_T _*) increases, the amplitude of the oscillation in the repressilator can be changed significantly (B and C). A repressilator model was obtained from the BioModels Database BIOMD0000000012 [50]. The model was modified to lower the expression levels (by changing translation efficiency to 10 and *K_M _*to 10 molecules per cell and the maximum transcription rate to 3 molecules per min per cell) and to add promoter binding-unbinding reactions for TetR repressors (for the detailed model description, refer to Additional File 1).

Predictable composition is of particular interest to the synthetic biology community where gene circuits are "wired" together via transcription factors (TFs) and corresponding promoters (Figure 1). This mode of wiring makes the physical construction of relatively complex networks possible [11,12]. However the general question of whether making a connection between two genetic units results in a predictable functional whole remains. In particular, a number of issues present themselves that include independence from the surrounding milieu (also called orthogonality [5]): domain matching and impedance bridging. The former describes the situation where the operating concentration range of an output transcription factor matches the range of the input target. Impedance bridging, which is the main topic of this paper, is concerned with how much a downstream target circuit can affect the functional properties of an upstream unit. It is not to be confused with impedance matching which is related to the maximum power transfer between two circuits. There has recently been interest in defining impedance bridging in genetic and protein circuits [13-15] and a related quantity called retroactivity was introduced by Saez-Rodriguez et al. [16] and Del Vecchio et al. [17] to describe the effect of one module on another.

In electrical engineering there exist guidelines and published constraints on how many electrical modules can be driven from a source. For example, one rule of thumb for analog circuits suggests that the impedance at the input should be ten times the impedance at the driving circuit. In digital circuits, such as TTL (transistor-transistor logic) circuits [18], manufacturers will quote the fan-out and fan-in for a given electrical module. The fan-out indicates how many downstream logic gates can be connected to a given output. Exceeding these limits will potentially cause signal distortion in analog circuits and circuit failure in digital circuits. We envision the development of similar criteria for connecting two biological modules together in synthetic biology and introduce the notion of fan-out for a genetic circuit. The fan-out of a genetic circuit is defined as the maximum number of downstream promoters that can be driven from an upstream circuit signal without significant time-delay or signal attenuation.

In this manuscript, an experimental estimation method for the fan-out is proposed. This method is based on a linear relationship between a certain property of a module (response time) and the amount of load from downstream components (e.g., the number of downstream promoters in gene circuits). This linear relationship has not been discussed in previous work, for example the work by Del Vecchio et al [17,19]. By taking into account the linearity, we extend the retroactivity concept to the fan-out. Our analysis shows that the linear relationship holds not only for the simple module interface that Del Vecchio et al. considered [17] but also for a much wider class of interface. The linear relationship is shown to provide a unifying way for evaluating the fan-out in an efficient manner for all the interfaces belonging to the class. The fan-out can be estimated by using the autocorrelation [20-25] of gene expression noise [26-29]. During the estimation procedure the system's retroactivity can also be measured. Although our analysis is focused on genetic networks, the principles apply equally to signal transduction networks.

## Results and Discussion

### Module interface process

When two synthetic gene circuits are connected, transcription factors are used to connect them. The reaction processes involving the transcription factors such as transcription, translation, degradation, and downstream-module promoter regulation, will be called *module interface processes *(MIPs). For example, consider the repressilator [30]. Let us choose the TetR repressor, one of the genes comprising the oscillator, as an output of the oscillator module (Figure 1). When a downstream module has *tetR*-operons, the MIP includes *tetR*-transcription, translation, and TetR binding/unbinding to its specific operons located in the downstream module.

### Retroactivity and mapping between a module interface process and an RC-circuit

We investigate a MIP by mapping it to a simple electric circuit composed of a resistor and a capacitor connected in series (RC circuit). This mapping becomes significantly helpful for understanding retroactivity [17] and quantifying fan-out.

#### Isolated case

**Figure 2:**
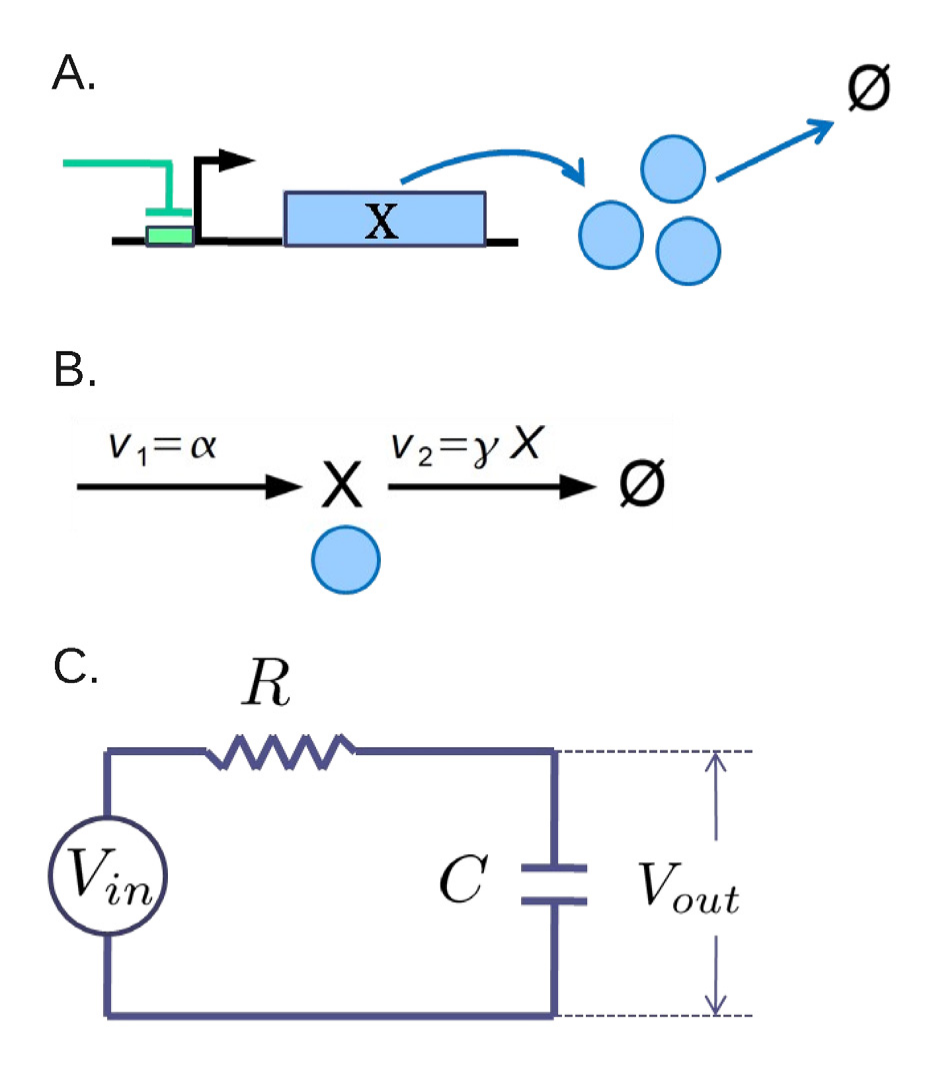
**Isolated module output**. Translation-degradation processes for *X *(A) can be described by a simple reaction process (B) with *α *a translation rate and *γX *a degradation rate. These processes can be mapped to an RC-circuit with *R *resistance and *C *capacitance by *V_out _*= *X*, *V_in _*= α/γ, and *RC *= 1/γ (C).

When an upstream output does not regulate any downstream promoter, the corresponding MIP can be modeled as a simple TF translation-degradation process (see Figure 2A and 2B). The concentration of the TF, denoted by *X*, changes in time by following the equation

(1) $$\frac{dX}{dt}=\alpha (t)-\gamma X,$$

with *α*(*t*) the translation rate and *γ *the degradation rate constant. We show how this process can be related to an RC circuit, where a resistor and capacitor are connected in series and driven by an input voltage source *V_in _*(Figure 2C). The total voltage drop across both the resistor and capacitor is equal to the driven voltage: *V_in _*= *RI *+ *V_out_*, where *I *denotes the current flowing through the resistor, and *V_out _*the voltage drop across the capacitor. The current is equal to the rate of charge accumulation (*Q*) in the capacitor: *I *= *dQ/dt*, where the small increment *dQ *causes the change in *V_out _*in proportion to *dQ*: *dQ *= *CdV_out_*, with *C *a proportionality constant called capacitance. Thus, the current *I *can be expressed as *CdV_out_/dt*. By substituting this into *V_in _*= *RI *+ *V_out _*and dividing the resultant equation by *RC*, we obtain

(2) $$\frac{dV_{out}}{dt}=\frac{V_{in}}{RC}-\frac{V_{out}}{RC},$$

where *RC *is known as the response time *τ*_0 _of the RC-circuit [31]. By comparing Eqs. (1) and (2), the following correspondence is obtained: *X *= *V_out_*, *α *= *V_in_/RC*, and γ = 1/*RC*, and the response time is expressed as

(3) $$\tau_{0}=RC=\frac{1}{\gamma }.$$

Thus, the TF-translation-degradation process (Figure 2B) can be directly mapped to the RC-circuit (Figure 2C).

#### Connected case

**Figure 3:**
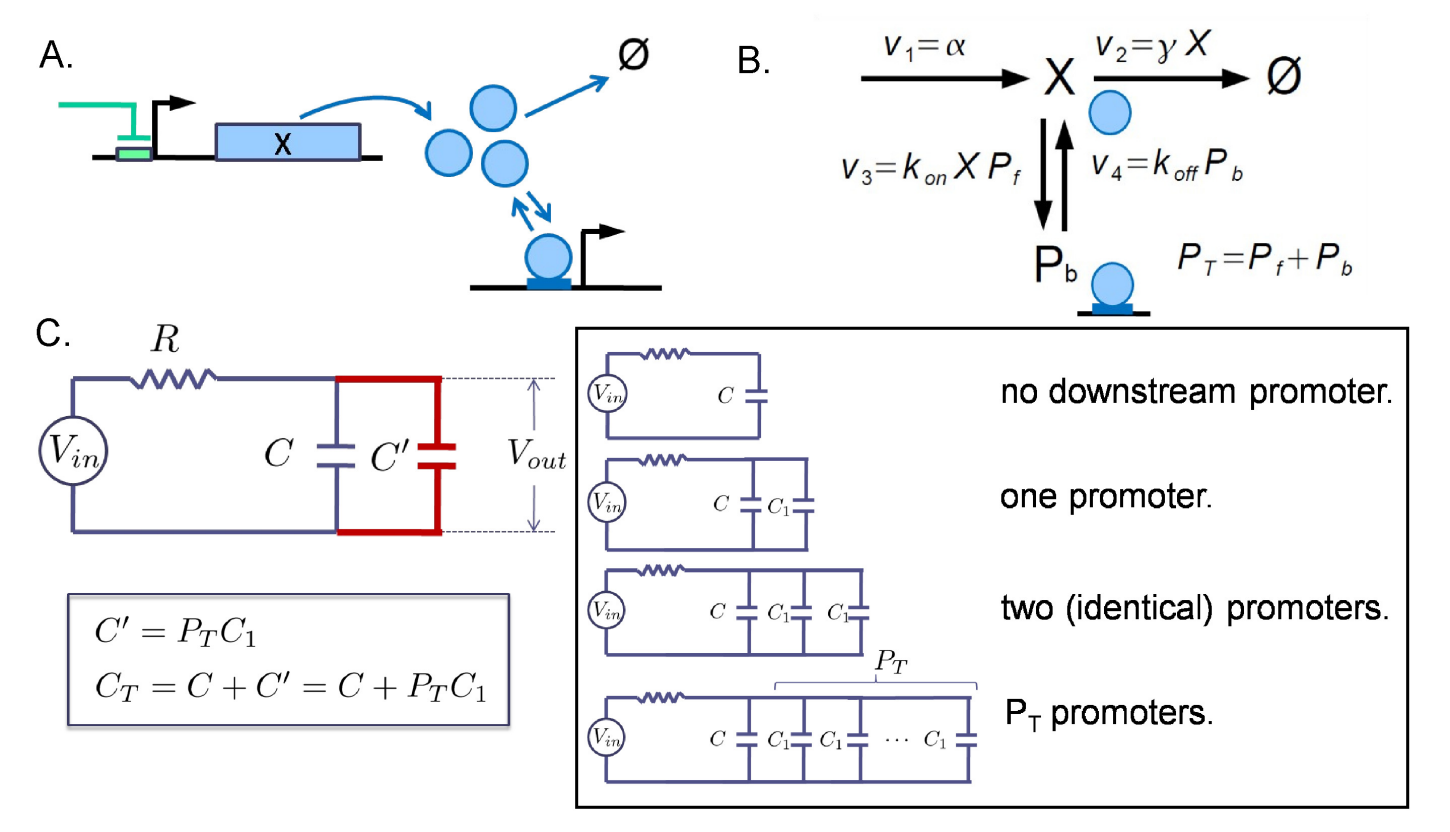
**Module interface process**. The output *X *of an upstream module regulates the downstream-module *X*-specific promoter (A). The translation-degradation processes for *X *and its promoter regulation can be modeled as the reaction process shown in B, where *P_f _*, *P_b_*, and *P_T _*denote the numbers of free, bound, and total promoters, respectively. The reaction process is mapped to an RC-circuit with an increased capacitance by *C'*, which is shown to be proportional to *P_T _*: *C' *= *P_T_C*_1 _with *C*_1 _a proportionality constant. This means that each promoter acts as a capacitor with a unit load of capacitance, *C*_1_. The total capacitance *C_T _*becomes the sum of the capacitance *C *in the isolated case and the extra capacitance *C'*.

When two modules are connected (see Figure 3A), Del Vecchio et al. [17] have shown that the interfacial dynamics slows down in response to interactions with downstream components. The degree of the slow-down defines the retroactivity as follows: The retroactivity describes a number between zero and one with one being the least desirable, i.e. the interfacial dynamics are affected most. In their analysis, they assumed that the binding-unbinding process of the TF is fast enough that the process can be approximated to be in the quasi-steady state (*k_on_X *+ *k_off _*≫ γ; cf. [32-34]). They also assumed that the lifetime of the bound TF is much longer than that of the unbound TFs.

Specifically, they showed that the free TF concentration *X *changes in time by the following equation [17]

(4) $$\frac{dX}{dt}=(1-ℛ(X))(\alpha -\gamma X),$$

where ℛ(*X*) is the *retroactivity*, given as

(5) $$ℛ(X)={\left[1+{\left(1+\frac{X}{K_{d}}\right)}^{2}\frac{K_{d}}{P_{T}}\right]}^{-1},$$

with *K_d _*the dissociation constant for the TF with respect to the promoter, and *P_T _*the total number of the promoters. They showed that ℛ is always less than 1 and non-negative. The extra factor 1 - ℛ appears when compared with the isolated case, resulting in the slow-down of the dynamics. More precisely, the slow-down is due to the decrease in the factor placed in front of *X *in Eq. (4): γ(1 - ℛ), which is related to the apparent response time:

(6) $$\tau_{a}\equiv \frac{1}{(1-ℛ)\gamma }.$$

We consider the MIP shown in Figure 3A and 3B under the same assumptions as given by Del Vecchio et al [17]. To understand the retroactivity by using an RC-circuit analogy, consider a circuit shown in Figure 3C. The total capacitance becomes the sum of the two capacitances: *C_T _*= *C *+ *C'*. Thus, the response time becomes *RC_T _*: *τ *= *RC_T _*. The change in the output voltage is governed by the same equation as in the isolated case except the capacitance *C *is replaced to *C_T _*:

(7) $$\frac{dV_{out}}{dt}=\frac{V_{in}}{RC_{T}}-\frac{V_{out}}{RC_{T}}=\left[1-\frac{C^{\prime }}{C+C^{\prime }}\right]\left[\frac{V_{in}}{RC}-\frac{V_{out}}{RC}\right].$$

By comparing Eqs. (4) and (7), the retroactivity is given by the relative ratio of the new capacitance:

(8) $$ℛ=\frac{C^{\prime }}{C+C^{\prime }},$$

and that the response time *τ *corresponds to *τ_a _*(Eq. (6)):

(9) $$\tau =RC_{T}=\frac{1}{(1-ℛ)\gamma }.$$

Connecting downstream promoters in the MIP is therefore shown to be equivalent to connecting extra capacitors in parallel with an existing capacitor in the RC-circuit. Due to these extra capacitors, the circuit takes a longer time to fully charge all the capacitors, resulting in the slow-down in the circuit response time. Biologically, the bound promoters act as a reservoir of potentially free TFs: Whenever there is a change in the number of the free TFs, the reservoir quickly buffers the change in the number of free TFs [35]. Such buffering causes transient dynamics at the interface to slow down.

### Response time vs. number of promoters

The response time is shown to increase with *P_T _*(see the Methods section) as

(10) $$\tau_{P_{\;T}}=R(C+P_{T}C_{1})\;,$$

where *C*_1 _is a proportionality constant satisfying *C/C*_1 _= *K_d _*(1 + *X/K_d_*)^2^. The above equation (10) can be viewed as each individual promoter contributing an extra capacitance *C*_1 _to the total capacitance (Figure 3C): *C_T _*= *C *+ *P_T_C*_1_. The capacitance *C*_1 _of each extra capacitor is related to a unit load onto the upstream output dynamics from a single downstream promoter. This is an interesting result and becomes useful for proposing an experimental method for estimating the fan-out.

The linear relationship between the extra capacitance and *P_T _*(see Figure 3C box) does not come from any linearization approximation, but from the fact that each downstream promoter affects the upstream as an independent effector (reservoir or sequestrator), although the sequestration itself is represented by a nonlinear reaction.

This linearity does not appear clearly in the retroactivity measure (Eq. (5)) proposed in [17], obscuring the connection to the concept of fan-out. The following section makes this connection and an efficient method for estimating the fan-out will be proposed.

### Gene circuit fan-out

A gene circuit fan-out is defined by the maximum number of promoters in a downstream module that the output (transcription factor) of an upstream module can regulate without altering the output dynamics significantly. To exemplify how much the upstream module can be affected, a repressilator [30] is considered as a module and its Tet repressors as a module output (Figure 1A). When the output regulates *tetR *promoters located in a downstream module, the oscillation amplitude of the *tetR *expression level can be significantly changed, e.g., 40% decrease when the number of the promoter (*P_T _*) is changed from 0 to 100 (Figure 1B and 1C). Our interest is here to quantify the maximum number of the promoters (fan-out) that the upstream module can tolerate.

**Figure 4:**
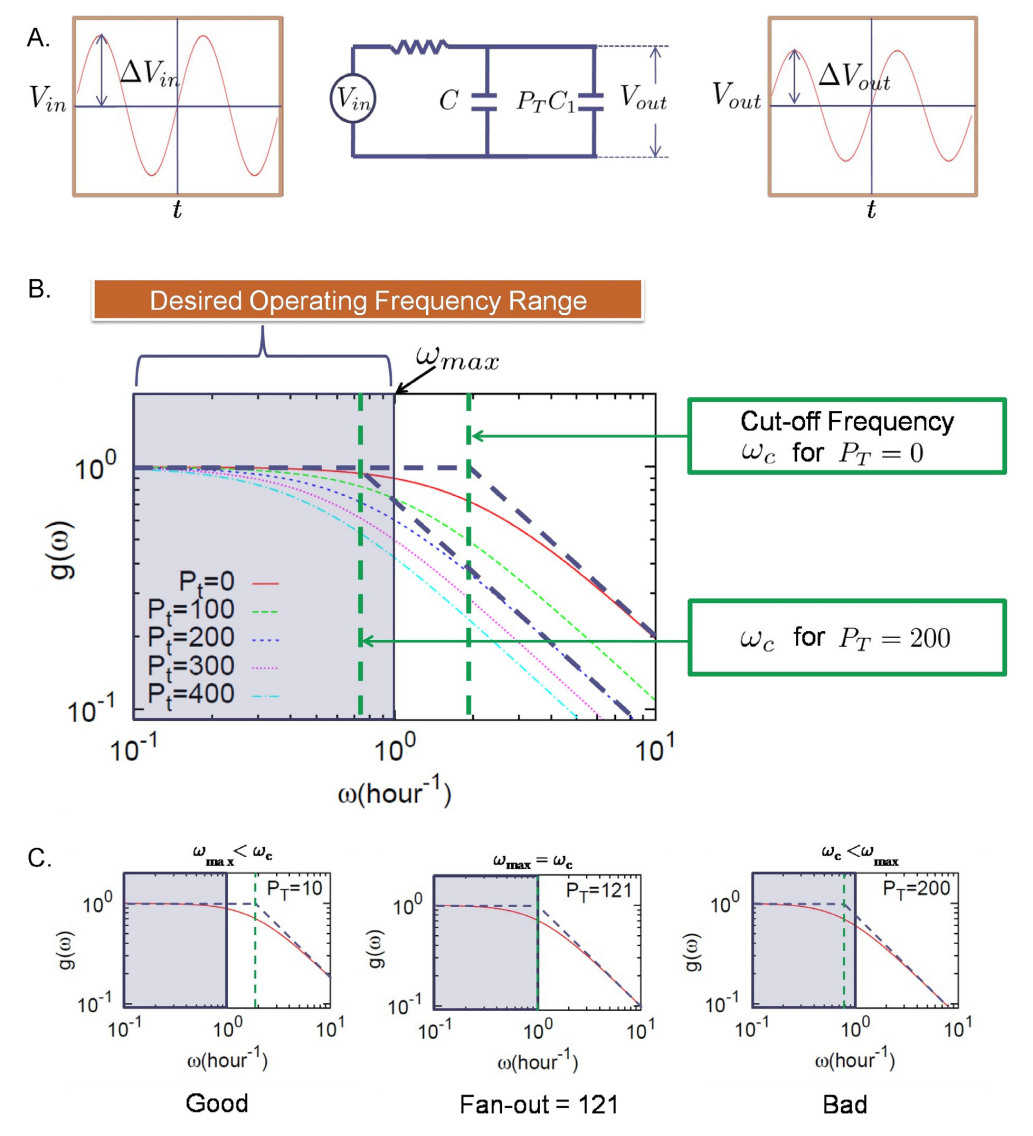
**Frequency response of the module interface process shown in Fig. 3A**. An oscillatory signal is applied at *V_in _*with different frequencies (A). The signal gain is defined by the ratio of the oscillation amplitude of the output signal (*V_out_*) to that of the input (Vin): $g\left(\omega \right)=\frac{\Delta V_{out}\left(\omega \right)}{\Delta V_{in}\left(\omega \right)}$, and can be well approximated by $g\left(\omega \right)={\sqrt{1+\omega^{2}/{\left(RC_{T}\right)}^{2}}}^{-1}$[31] with *C_T _*the total capacitance. As the number of the promoters that the output (TF) signal drives (regulates) increases, the cut-off frequency (*ω_c _*= 1/*RC_T _*) decreases (B). The output signal is desired to be operated within a certain frequency range, e.g., between 0 and 1 hour^-1^. Here the maximum operating frequency *ω_max _*is 1 hour^-1^. When the cut-off frequency matches the maximum operating frequency, the corresponding number of the promoters is defined as the fan-out (C). Parameters of the model: *K_d _*= 1 nM [*k_on _*= 10(1/nM/hour), *k_off _*= 10 (1/hour)], γ = 2(1/ hour), *α *= 20(nM/hour). Here the volume of a host cell is assumed roughly equal to 1 *μ*m^3^. Under the assumption, a copy number of one corresponds to 1 nM.

Let us quantify the fan-out by considering again the simple MIP shown in Figure 3A. We consider a frequency response between the input and output voltage, *V_in _*and *V_out_*, respectively (Figure 4A). In the RC circuit, the capacitor acts as a low pass filter: The gain of the signal (the ratio of the oscillation amplitude of *V_out _*to that of *V_in_*) is at the maximum level for low frequencies and drops significantly when the circuit no longer responds as fast as the input signal changes (Figure 4B). The frequency when this happens is called the *cut-off frequency *(*ω_c_*) (Figure 4B) and corresponds to the inverse of the response time: 1/*RC_T _*[31]. The cut-off frequency corresponds to the bandwidth in the low-pass filter [31], which defines the range of frequency where a signal gain is sufficiently large.

If the upstream module functions as a synthetic oscillator, there will be a practical upper limit (*ω_max_*) in the oscillator's frequency (e.g., for the repressilator, *ω_max _*can be the inverse of the repressor lifetime = log(2)/10 min^-1 ^~ 4 hour^-1 ^[30]). If *ω_max _*is smaller than the cut-off frequency *ω_c_*, the oscillator output will operate in a predictable manner and the output signal will be passed downstream without any significant signal loss. As the number of the downstream promoters increases, the total capacitance increases as shown in Eq. (10) and the cut-off frequency (*ω_c _*= 1/*RC_T _*) decreases. For the cut-off frequency to be larger than the maximum operational frequency *ω_max_*, the total number of the promoter must be smaller than a certain value, which will be called the *fan-out*. The fan-out denoted by $F_{\omega_{max}}$ is obtained where *ω_c _*equals *ω_max_*, i.e., $\omega_{c}={\left[R\left(C+P_{T}C_{1}\right)\right]}^{-1}=\omega_{max}$:

(11) $$F_{\omega_{max}}=\frac{C}{C_{1}}\left[\frac{1/\tau_{0}}{\omega_{max}}-1\right].$$

In the fan-out equation (11), there are two unknown parameters: *C/C*_1_, and *τ*_0_. These can be experimentally estimated by performing two independent experiments with and without any downstream module. In each experiment, the corresponding response time, *τ*_0 _or $\tau_{P_{T}}$, can be estimated (by using gene expression noise as will be presented later in the Results section). Thus, one of the unknowns *τ*_0 _can be estimated. How can the other unknown *C/C*_1 _be estimated from $\tau_{P_{T}}$? If the copy number of the promoters *P_T _*is known a priori, the value of *C/C*_1 _can be obtained from Eq. (10). If the promoters are placed on plasmids, the copy number of the plasmids can be estimated depending on what type of origin of replication is used, and thus the copy number of the promoters *P_T _*can be known. By calculating *τ*_0 _= *RC*_1_, the other unknown, *C/C*_1_, can be obtained.

### Gene circuit fan-out in more general interfaces

Up to now we have considered a simple MIP without feedback and where the degradation rate is assumed to be first-order. Here the more general case is considered and it is shown that the same or a similar fan-out function as Eq. (11) is obtained.

#### Oligomer under directed degradation and self-regulation

**Figure 5:**
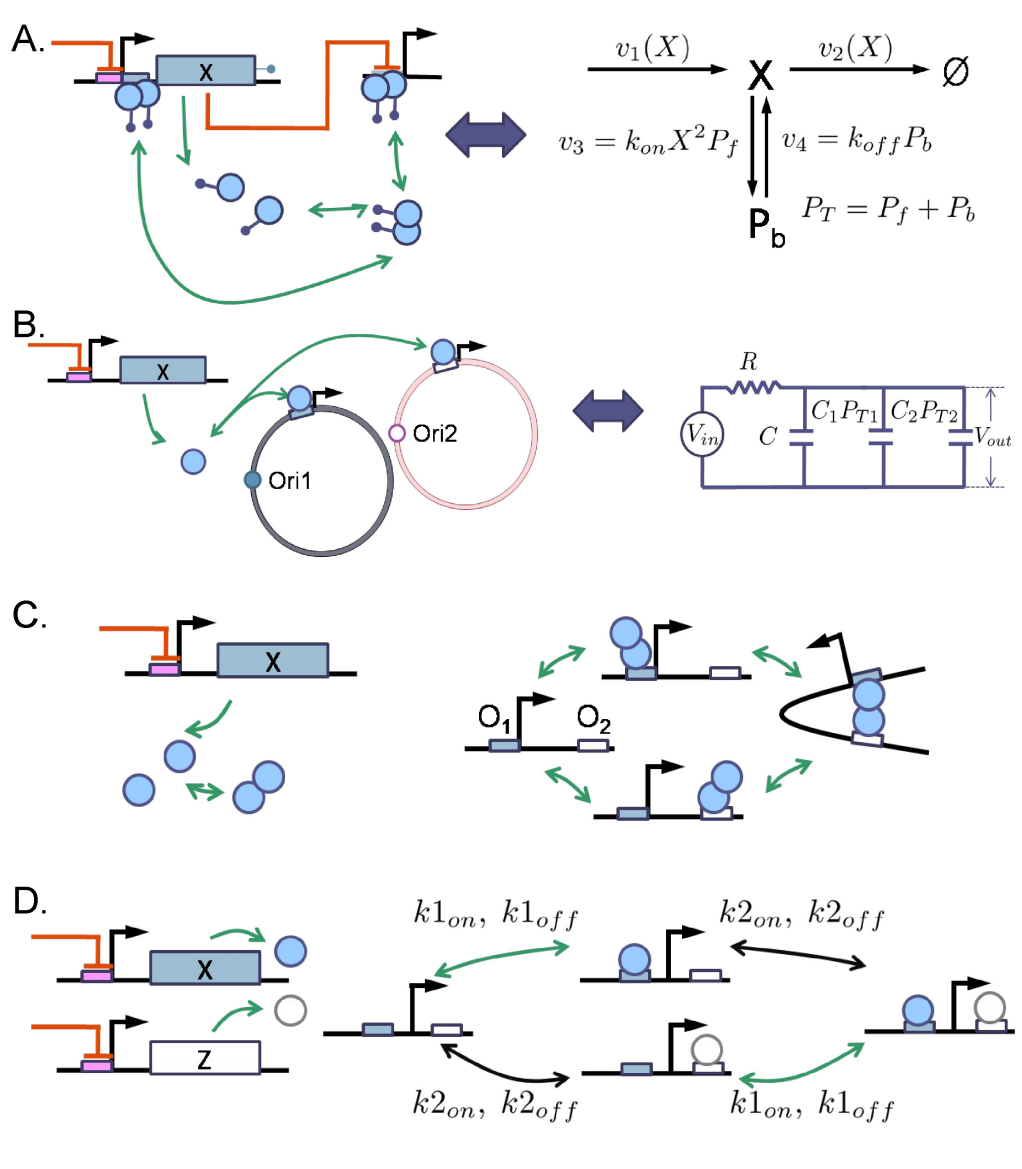
**Module interface processes that the fan-out functions Eqs. (11) and (12) can be applied to**. (A) An oligomer TF is degraded by proteases. (B) A TF can bind two different promoter plasmids having different binding affinities and different origins of replication. This can be mapped to an RC-circuit with two different capacitances connected in parallel. (C) An Oligomer TF can bind multiple operators. (D) Each different TF binds to its specific operator without affecting the binding affinity of the other.

Consider that a TF, composed of *n *monomers, is tagged for degradation and that its transcription is self-regulated as shown in Figure 5A. The fan-out function is obtained by the same equation (11), where *τ*_0 _is the time constant in the isolated case, given by the difference between the unscaled elasticities [36]: 1/*τ*_0 _= *ε*_2 _- *ε*_1_, where *ε*_1 _≡ ∂*v*_1_(*x,α*)/∂*x *and *ε*_2 _≡ ∂*v*_2_(*x*)/∂*x *(refer to the Methods section). This means that the fan-out can be estimated exactly in the same way as in the monomer case as shown in Figure 3A. All the above results apply for the case that the *intermediate *reaction steps of the oligomerization and directed degradation are taken into account (refer to Additional File 1 and the Example 2).

#### Multiple promoters having different affinities

Consider the case that two different types of TF-specific promoter plasmids, having different affinities for the TF and different strength of the origin of replication. It is shown that the MIP can be mapped to an RC-circuit having two different capacitances connected in parallel to *C *as shown in Figure 5B (see the Methods section). The fan-out of each promoter is shown to satisfy the following functional relationship between *F*_1 _and *F*_2 _(refer to the Methods section):

(12) $$1/\omega_{max}=\tau_{0}\left(1+F_{1}\frac{C_{1}}{C}+F_{2}\frac{C_{2}}{C}\right),$$

where *F_i _*is the fan-out for promoter plasmids of the *i*-th kind, and *C_i _*denotes the corresponding capacitance per plasmid. If there are *N *different kinds of promoter plasmids, all the *N *capacitances need to be summed up in the above equation. Here the fan-out is not a single number but is given by a functional relationship between *F_i_*'s: The number of plasmids of different kinds needs to be balanced depending on its unit load on the retroactivity, i.e., *C_i_/C*.

To obtain the fan-out function, it is necessary to find three unknown parameters: *τ*_0_, *C*_1_/*C*, and *C*_2_/*C*. *τ*_0 _can be estimated in the isolated case. *C_i_/C *can be estimated in the case that only the *i*-th kind of promoter plasmids exists (under the assumption that the strength of each origin of replication is already known). These three independent experiments will suffice for estimating all the unknown parameters and proposing the fan-out function Eq. (12).

#### Multiple operators

Consider the case that the promoter region includes multiple operators specific to an output TF (e.g., *O*_1_, *O*_2_, and *O*_3_) having different affinities (Figure 5C). Regardless the number of the operators, the same fan-out function as Eq. (11) is obtained (refer to Additional File 1).

#### Two output signals

When two output TFs (*X *and *Z*) regulate a downstream promoter independently, i.e., if there is no overlap between the operator regions and somehow *X *does not interfere with the operator region of *Z *and vice versa, the fan-out corresponding to each output TF can be obtained.

The fan-out functions like Eqs. (11) and (12) have been shown for each of the individual cases given above. For all the combinations of these individual cases the same fan-out functions will apply as well.

### Design scheme for fan-out enhancement

**Figure 6:**
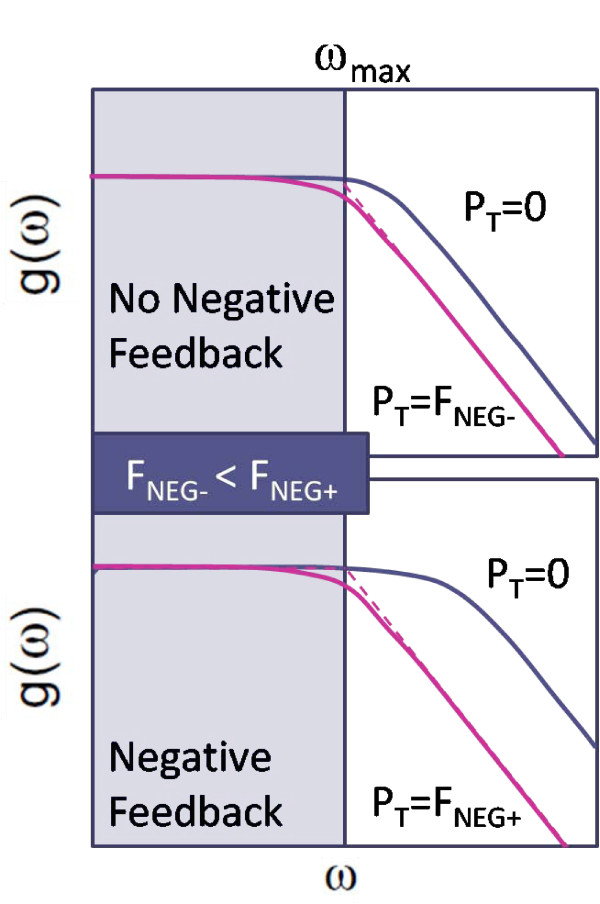
**Fan-out enhancement due to negative feedback**. One of the mechanisms to enhance fan-out is to apply negative feedback on the output TF. This pushes the cut-off frequency away from the maximum desired operating frequency (*ω_max_*), resulting in a larger value of fan-out: *F*_*NEG+*_>*F*_*NEG-*_, where *F_NEG+ _*and *F_NEG- _*are the values of fan-out with and without negative feedback, respectively.

How can we increase the fan-out? Based on the fan-out equations (11), there are two ways: increasing *C/C*_1 _or 1/*τ*_0_. The way to increase the latter is to apply a negative feedback on the translation of *X *(making *ε*_1 _negative for the case shown in Figure 5A, where 1/*τ*_0 _= *ε*_2 _- *ε*_1_) and a positive feed-forward on the degradation rate (increasing *ε*_2_). These applications push the cut-off frequency/bandwidth (1/*τ*_0_) further away from the maximum desired operating frequency (*ω_max_*), and enhances the fan-out as illustrated in Figure 6. A simulation study of enhancing fan-out will be presented later in this manuscript. Since the enhanced degradation and negative feedback decrease the concentration level of *X*, to prevent this, it is desirable to amplify the translation rate (which makes *ε*_1 _more negative).

This mechanism for enhancing fan-out is exactly the one proposed by Del Vecchio et al. [17] to reduce retroactivity; when the retroactivity is small, the upstream output dynamics does not slow down significantly by connecting the output to the downstream module, meaning that the load from downstream to the upstream is small enough that many replicates of the load can be applied to the upstream without slowing down the output dynamics significantly.

One of the mechanisms, inhibitory auto-regulation, is frequently found in *Escherichia coli *transcription factors regulating a set of operons, e.g., for amino-acid biosynthesis where a single TF may control multiple targets, likewise for flagella formation [37]. Such motifs are called single-input-module motifs [37].

The concept of fan-out is not limited to gene regulatory circuits. In principle, as long as the same class of interface processes are found regardless of the type of biological systems, the fan-out and retroactivity concepts can be applied [1,17]. For example, in the eukaryotic MAPK pathway, doubly phosphorylated MAPK can activate a number of downstream proteins and transcription factors in the nucleus. This MAPK regulation can be described by the module interface process similar to the one shown in Figure 5B (in this case, many promoter plasmids instead of the two). In the MAPK pathway, there is a negative feedback from MAPK to the phosphorylation of MAPKKK [13-15]. In a recent paper by Yu et al. [38] which showed experimentally that the related system in Yeast involving Fus3 as the negative feedback component showed linearity between receptor occupancy and downstream response in the presence of feedback. Although they did not show increased fan-out *per se*, the presence of linearity may suggest there is an increase in fan-out of the MAPK module, thereby permitting MAPK to effectively regulate multiple targets and multiple homologous binding sites.

### How to measure the time constant *τ*

It is known that transcription factors show significant stochastic fluctuations [22-25,39-42] (for review articles, [26-29]). Their correlation times have been measured by obtaining autocorrelations by *in vivo *time-lapse microscopy [22-25]. Recent numerical studies show that the correlation time is approximately equal to the response time of the deterministic case [43] and that it changes as a result of connecting two genetic systems [19,43]. Therefore, from the change in the correlation time, the fan-out can be estimated by using Eq. (11) as well as the retroactivity by using $ℛ=\frac{\tau_{P_{T}}-\tau_{0}}{\tau_{P_{T}}}$ (obtained from Eq. (8) by using Eqs. (3) and (9)).

### Example 1: Fan-out/retroactivity estimation

**Figure 7:**
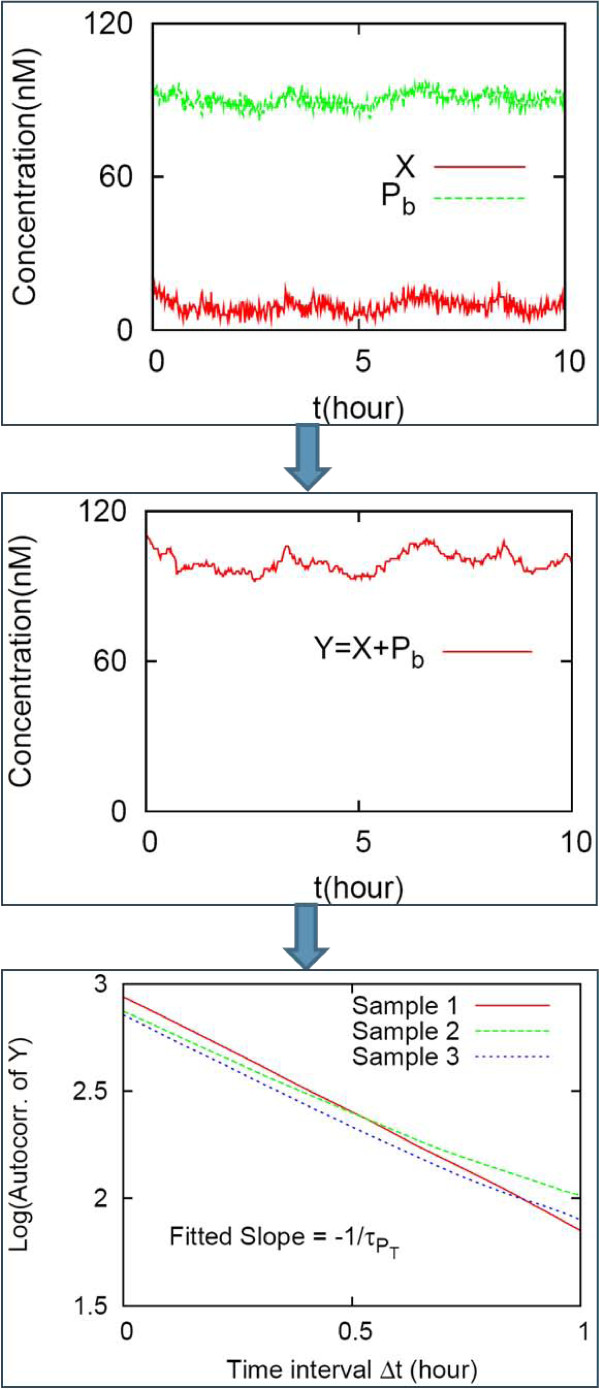
**Estimation of response time constants from autocorrelation functions**. The concentrations of free and bound TFs (*X *and *P_b _*respectively) fluctuate stochastically for the MIP shown in Fig. 3A. The total TF concentration (*X *+ *P_b_*) can be observed experimentally; for example, when the TF is tagged for fluorescence, the fluorescence intensity could reflect the total TF concentration [43]. The autocorrelation of the total is fitted to an exponential function: A linear fit is taken in the semi-log scale. The inverse of the fitted slope corresponds to the response time constant $\tau_{P_{T}}$. The error bar of the $\tau_{P_{T}}$ are estimated from 10 independent replicates of autocorrelation functions (only three samples are shown in the figure). The same parameter values are used as in the Fig. 4 caption and the value of *P_T _*is set to 100.

In this example, the simple MIP shown in Figure 3A is considered as a model for TFs in *E. coli*. The average copy number of plasmids containing the specific promoters is assumed to range from 1 to 100. The volume of *E. coli *is assumed to be roughly equal to 1 *μ*m^3^, and a copy number of one corresponds to 1 nM. As a result, the unit of nM is henceforth interchanged with that of copy number. A simulation using the standard Gillespie method [44] was performed (see Figure 7) and the observed autocorrelation was fitted to an exponential function: *G*(Δ*t*) = *A *exp (-Δ*t*/*τ*) with *τ *a correlation time (a linear fit is conducted in the log-scale in the *y*-axis and the normal scale in the *x*-axis) and 1/*τ *obtained from the fitted slope (see Figure 7).

For experimentally reasonable parameter values, i.e., *α *= 20 nM hour^-1^, γ = 2 hour^-1^, *k_on _*= 10 nM^-1^hour^-1^, and *k_off _*= 10 hour^-1^, stochastic simulations were performed with and without any downstream-module promoter (*P_T _*= 100 and 0). The concentration levels of the total TF was recorded for 48 hours (corresponding to experimental time) with frequency 50 times per hour, the autocorrelation of this signal was fitted to an exponential function, and the response time measured (see Figure 7) [43]. The error bar of the time constant was obtained from 10 independent replicates of the autocorrelation.

When the translation rate was set to 20 nM hour^-1^,*τ*_0 _and *τ*_100 _were obtained to be 0.52 ± 0.06 hour and 0.9 ± 0.1 hour, respectively. The value of *C/C*_1 _was obtained to be 140 ± 20, by using

(13) $${\frac{C}{C_{1}}=P_{T}\frac{RC}{RC_{T}-RC}=P_{T}\frac{\tau_{0}}{\tau_{P_{T}}-\tau_{0}}|}_{P_{T}=100},$$

where *C_T _*- *C *= *P_T_C*_1 _was used. From Eq. (11), the fan-out function for this MIP was obtained:

(14) $$F_{\omega_{max}}=140\left[\pm 20\right]\left(\frac{1/0.9[\pm 0.1]}{\omega_{max}}-1\right).$$

If the upstream module is a synthetic oscillator with a maximum operating frequency *ω_max _*= 1 hour^-1^, the fan-out becomes *F *= 130 ± 20. This means that promoter plasmids with low, medium, and high copy numbers can be used without affecting the TF dynamics, if a single TF-specific operator site resides on a plasmid. The retroactivity can also be estimated from the measured values of *τ*_0 _and *τ*_100_: ℛ = 0.4 ± 0.1.

If the translation rate is reduced by half (now,*α *= 10 nM hour^-1^), the free TF concentration decreases by half. As the concentration decreases, the retroactivity increases [17,43] and the fan-out decreases. The values of *τ*_0 _and *τ*_100 _are obtained to be 0.52 ± 0.07 hour and = 1.75 ± 0.04 hour, respectively. For the same *ω_max _*= 1 hour^-1^, the fan-out becomes *F *= 40 ± 1. This would mean that only low copy number plasmids can be safely used. The retroactivity is estimated to be 0.70 ± 0.05.

### Example 2: Fan-out enhancement by applying negative feedback

In this example, the MIP of dimer TFs is considered that are under inhibitory self-regulation as shown in Figure 5A. The reaction process can be described by the following set of reactions:

$$\begin{array}{c} \overset{\frac{\alpha }{1+\beta X_{2}}}{\to }X\;\overset{\gamma X}{\to }\emptyset \\ X+X\overset{k_{1}X(X-1)}{\underset{k_{2}X_{2}}{⇌}}X_{2}\overset{\gamma_{2}X_{2}}{\to }\emptyset \\ X_{2}+P_{f}\overset{k_{on}X_{2}P_{f}}{\underset{k_{off}P_{b}}{⇌}}P_{b}, \end{array}$$

where *β *is introduced to turn on and off the negative feedback. The following parameter values are used: *α *= 20(nM/hour), γ = 2(1/hour), *k*_1 _= 20(1/nM/hour), *k*_2 _= 1(1/hour), γ_2 _= 2(1/hour), *k_on _*= 10(1/nM/hour), and *k_off _*= 10(1/hour) for the case without any feedback (*β *= 0). For the case with negative feedback (β = 0.25), the value of *α *was adjusted to match the same expression level of *X *as the case without feedback: *α *= 43. The data are sampled in the identical way as described in the Example 1 and the response time constants were measured from the autocorrelations of the total TF concentrations (*X *+ 2*X*_2 _+ 2*P_b_*).

**Figure 8:**
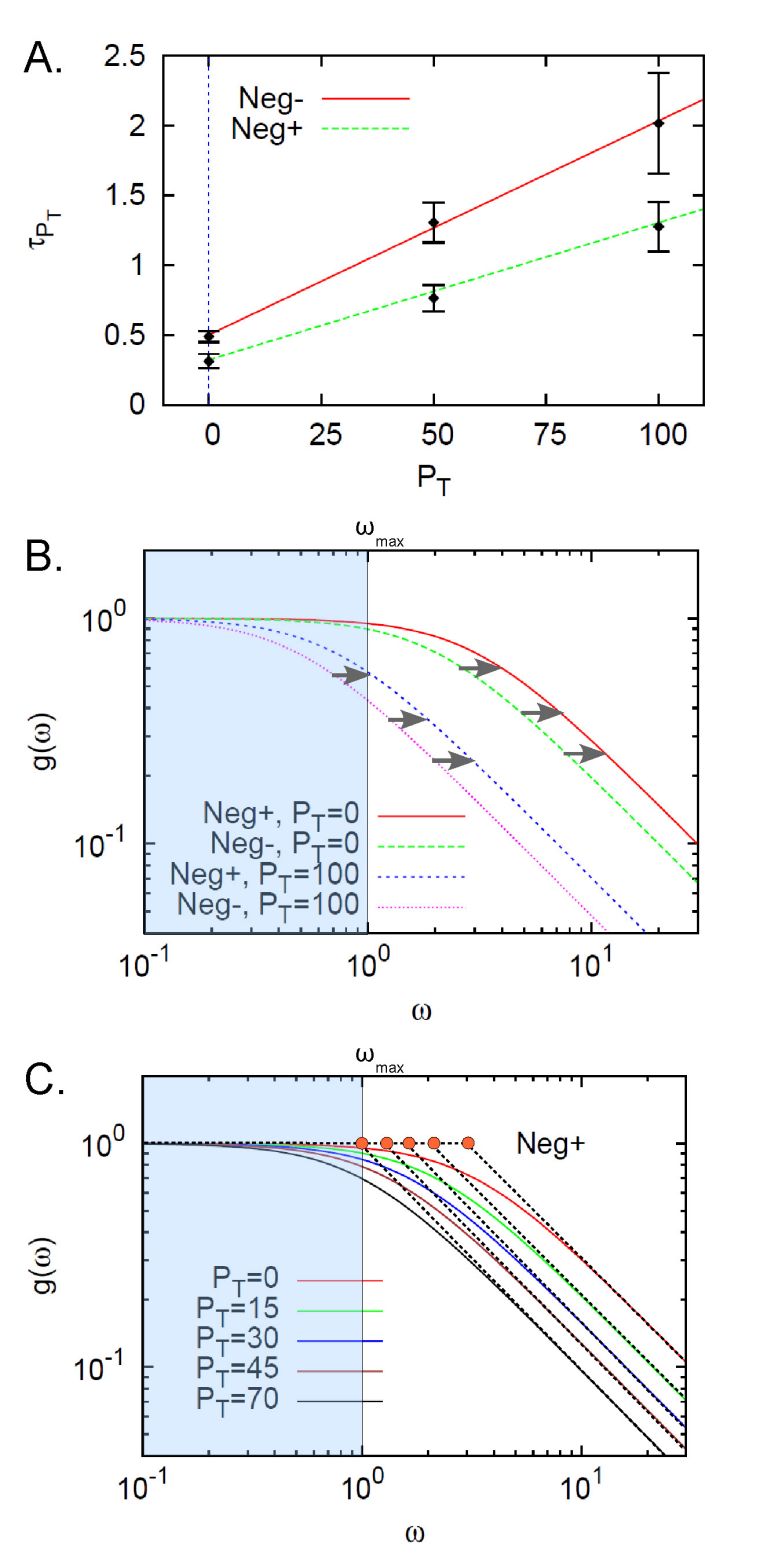
**Estimation of fan-out from the measured response time constants**. The module interface process involving dimer TFs is considered when the TF-expression is under inhibitory self-regulation (shown in Fig. 5A and reaction process (14)). (A) The response time constant $\tau_{P_{T}}$ of the transcription factor increases linearly with the number of the downstream promoters *P_T _*. The time constants were measured from the autocorrelation functions of the concentration levels of the total transcription factors (*Y *= *X *+ 2*X*_2 _+ 2*P_b_*). The measured time constants are consistent with the deterministic prediction (lines) and are used to obtain the slope and y-intercept that are *RC*_1 _and *RC*(=*τ*_0_), respectively, by performing a linear fit. From the obtained *RC*_1 _and *RC*, the fan-out function is estimated. (B) The normalized signal gain of the output signal *Y *with respect to the input signal *α *(translation rate) is plotted for different frequencies. With negative feedback, the bandwidth increases. (C) The increased bandwidth results in the fan-out enhancement.

Figure 8A shows that $\tau_{P_{T}}$ estimated from the autocorrelations linearly increases with *P_T _*while satisfying the deterministic prediction based on Eq. (10), which was computed by using Mathematica [45] (its notebook file is provided in Additional File 2). The value of *C/C*_1 _was estimated from both the slope (*RC*_1_) and y-intercept (*τ*_0 _= *RC*) of the graph shown in Figure 8A: *RC*_1 _= 0.016 ± 0.002 hour and *τ*_0 _= 0.49 ± 0.04 hour for the case without feedback. For *ω_max _*= 1 hour^-1^, the fan-out *F_Neg- _*was estimated to be 32 ± 7. For the case with feedback, the values of *RC*_1 _and *RC *were obtained to be 0.009 ± 0.001 hour and 0.31 ± 0.05 hour, respectively. The fan-out *F_Neg+ _*was estimated to be 77 ± 15. With negative feedback, the fan-out was increased two-fold.

This fan-out enhancement can be understood in terms of the cut-off frequency increase. The signal gain in the output signal (total transcription factor) with respect to the input (translation rate) was plotted for different frequencies as shown in Figure 8B and 8C, and the cut-off frequency was shown to increase due to the negative feedback and to push the cut-off frequency away from the maximum desired operating frequency. This allows for a larger load from the downstream, resulting in the enhanced fan-out.

### Effect of cell's machinery on fan-out

**Figure 9:**
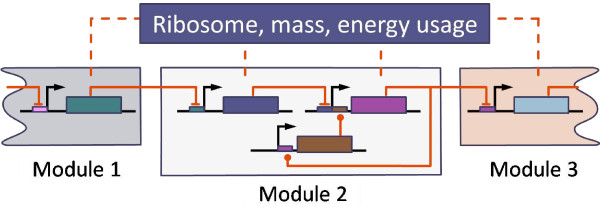
**Effect of cell's machinery on fan-out**. The fan-out of Module 1's output can be affected by downstream Modules 2 and 3 via the cell's machinery (ribosomes, mass and energy usage).

What we have not considered in this paper is the important case of cascading gene regulatory circuits (Figure 9). The question that arises here is whether the fan-out and retroactivity is affected in these situations. The answer depends on whether the transcription factors impose a significant load on the cell's machinery (ribosomes, mass and energy usage) or not. If not, then the fan-out values are unaffected because there is no upstream information transmission from downstream transcription factors. However, if the load on the cellular machinery is considered, then implied feedback and feed-forward loops appear, and this will have an effect on both the individual and net fan-out measures (Figure 9). This important topic is, however, beyond the scope of this paper and will be considered in a later publication.

## Conclusions

In this paper, the concept and quantitative measure of fan-out have been introduced for genetic circuits. The fan-out is a measure of the maximum number of promoter sites that the output TFs of the upstream module can regulate without significant slow-down in the kinetics of the output. In addition, an efficient experimental method to estimate the fan-out have been proposed. The fan-out has been shown to be enhanced by self-inhibitory regulation on the output. In the estimation process of the fan-out, the retroactivity can also be computed. This study provides a way for quantifying the level of modularity in gene regulatory circuits and helps characterize and design module interfaces and therefore the modular construction of gene circuits.

## Methods

This section shows the mathematical derivations of the time-constant $\tau_{P_{T}}$ and fan-out functions for various cases.

### Monomer TF

Consider the simple MIP shown in Figure 3. Under the quasi-equilibrium in the binding-unbinding process, the concentration of the bound TF is obtained as *P_b _*= *P_T_X*/(*X*+*K_d_*). This equation is simplified to, by introducing *f*(*X*) ≡ *X/*(*X *+ *K_d_*),

(15) $$P_{b}\text{=}P_{T}f\left(X\right)\text{.}$$

The time evolution of the total transcription factor (*Y *= *X *+ *P_b_*) is governed by the following equation [43]:

$$\frac{dY}{dt}=\alpha -\gamma X.$$

The response time constant of *Y *is derived rather than that of *X*, because *Y *is a pure slow variable showing the dynamics of our interest [17,33,43]. The response time constant of *Y*, denoted by $\tau_{P_{T}}$, is obtained by taking the derivative on the right hand side of the above equation with respect to *Y *:

(16) $$\tau_{P_{T}}=-{\left[\frac{d\left(\alpha -\gamma X\right)}{dY}\right]}^{-1}=\tau_{0}\frac{dY}{dX},$$

with *τ*_0 _≡ γ^-1^. By using *Y *= *X *+ *P_b_*, the above equation becomes $\tau_{P_{T}}=\tau_{0}\left[1+\frac{dP_{b}}{dX}\right]$. By using Eq. (15), the time constant is obtained: $\tau_{P_{T}}=\tau_{0}\left(1+f^{\prime }\left(X\right)P_{T}\right)$. By comparing this with Eq. (10), *C/C*_1 _becomes *K_d_*(1 + *X/K_d_*)^2^.

### Oligomer TF under directed degradation and self-regulation

Consider that the transcription factors are composed of *n *monomers described in Figure 5A. The binding-unbinding process is assumed to be in equilibrium, and *P_b _*is obtained as

$$P_{b}=\frac{P_{T}X^{n}}{X^{n}+K_{d}}\equiv P_{T}f\left(X\right).$$

The time evolution of *Y *(= *X *+ *nP_b_*) is governed by

$$\frac{dY}{dt}=v_{1}\left(X\right)-v_{2}\left(X\right).$$

The response time constant of *Y *is obtained as

$$\tau_{P_{T}}=-{\left[\frac{d\left[v_{1}\left(X\right)-v_{2}\left(X\right)\right]}{dY}\right]}^{-1}=\tau_{0}\frac{dY}{dX},$$

where *τ*_0 _≡ 1/(*ε*_2 _- *ε*_1_) with *ε*_1 _≡ *∂v*_1_/∂*X *and *ε*_2 _≡ *dv*_2_(*X*)/*dX*. By using *Y *= *X *+ *nP_b_*, the response time is obtained as

$$\tau_{P_{T}}=\tau_{0}\left[1+P_{T}\;n\frac{df\left(X\right)}{dX}\right],$$

Where $n\frac{df(X)}{dX}$ is defined to be *C*_1_/*C *by comparing the above equation with Eq. (10).

### Multiple promoters having different affinities

We consider the case that two different types of TF-specific promoter plasmids, having different affinities for the TF and different strength of the origin of replication. The TF is assumed to be a monomer. The concentration of the TF bound on the promoter of each type (*i*) is given as $P_{bi}=\frac{P_{Ti}X}{X+K_{di}}\equiv P_{Ti}f_{i}\left(X\right)$ for *i *= 1, 2. The response time constant $\tau_{P_{T1},P_{T2}}$ is given by Eq. (16). By using *Y *= *X *+ *P*_*b*1 _+ *P*_*b*2_, $\tau_{P_{T1},P_{T2}}$ becomes $\tau_{0}\left[1+\frac{dP_{b1}}{dX}+\frac{dP_{b2}}{dX}\right]$, which is rewritten as $\tau_{P_{T1},P_{T2}}=\tau_{0}\left[1+{f^{\prime }}_{1}\left(X\right)P_{T1}+{f^{\prime }}_{2}\left(X\right)P_{T2}\right]$ by using *P_bi _*= *P_Ti_f_i_*(*X*). Finally by equating $\tau_{P_{T1},P_{T2}}$ to 1/*ω*_*max*_, Eq. (12) is obtained. If there were *n-*different types of promoter plasmids, the above equation is changed by replacing the last two terms to the sum over all the *n*-types.

In the RC-circuit representation, $\tau_{P_{T1},P_{T2}}$ is given by *RC_T _*and the total capacitance *C_T _*is obtained as *C_T _*= *C *+ *C*_1_*P*_*T*1 _+ *C*_2_*P*_*T*2 _with $C_{i}=\tau_{0}{f^{\prime }}_{i}(X)/R$. This indicates that this MIP can be mapped to an RC-circuit having two different capacitances connected in parallel to *C *as shown in Figure 5B.

### Software

All stochastic simulations were carried out using our own Gillespie code written in C and run on a Quad-core PC under the Ubuntu Linux OS. Certain frequency plots, deterministic simulations and SBML translation of models to Jarnac script [46,47] were carried out using SBW [48,49].

## Competing interests

The authors declare that they have no competing interests.

## Authors' contributions

KHK primarily developed and carried out the proposed work. HS participated its design and provided critical feedback and suggestions. All authors read and approved the final manuscript.

## Supplementary Material

Additional file 1 **Supplementary document**. Description of the repressilator BioModel, and mathematical derivation of a linear relationship between *C' *and *P_T _*for individual cases of oligomer TFs (with and without negative feedback) and degradation-tagged TFs. Click here for file

Additional file 2 **Mathematica notebook**. Computation of fan-out values and frequency responses for the case of oligomer TFs that are under inhibitory self-regulation. Click here for file
