# Supplementary material for: Fan-out in gene regulatory networks
Source: J Biol Eng. 2010 Dec 17;4:16. doi: 10.1186/1754-1611-4-16 (PMC3024275; doi:10.1186/1754-1611-4-16)
Supplement: Additional file 1 — Supplementary document. Description of the repressilator BioModel, and mathematical derivation of a linear relationship between C' and PT for individual cases of oligomer TFs (with and without negative feedback) and degradation-tagged TFs. [file 1754-1611-4-16-S1.PDF]

Supplementary Information: Fan-out in Gene Regulatory Networks

Kyung Hyuk Kim *

Herbert M. Sauro †

Department of Bioengineering, University of Washington, Seattle, WA 98195, USA

S1 Repressilator BioModel

A model for the repressilator was obtained from the BioModels Database (model id: BIOMD0000000012) [1]. This model is composed of transcription and translation processes for respective *lacI*, *tetR*, and *cI*. Inhibitory regulations among them are described by Hill functions. One of the repressors, TetR, is chosen as an output of the repressilator module. The TetR repressors are allowed to regulate a downstream module and its regulation is described by the binding-unbinding reactions between the TetR and its specific promoter located in the downstream module. The copy numbers of mRNAs of respective *lacI*, *tetR*, and *cI* are denoted by X, Y, and Z, and the corresponding repressor molecules by PX, PY and PZ. The model process (per cell) is described as,

$$\xrightarrow{a_{0tr}+a_{tr}\frac{K_M^2}{K_M^2+PZ^2}} X \xrightarrow{kd_{mRNA}X} \emptyset,$$

$$\xrightarrow{a_{0tr}+a_{tr}\frac{K_M^2}{K_M^2+PX^2}} Y \xrightarrow{kd_{mRNA}Y} \emptyset,$$

$$\xrightarrow{a_{0tr}+a_{tr}\frac{K_M^2}{K_M^2+PY^2}} Z \xrightarrow{kd_{mRNA}Z} \emptyset,$$

$$\xrightarrow{k_{tl}X} PX \xrightarrow{kd_{prot}PX} \emptyset,$$

$$\xrightarrow{k_{tl}Y} PY \xrightarrow{kd_{prot}PY} \emptyset,$$

$$\xrightarrow{k_{tl}Z} PZ \xrightarrow{kd_{prot}PZ} \emptyset,$$

$$2PY + P_f \underset{k_{off}P_b}{\overset{k_{on}PY^2P_f}{\rightleftharpoons}} P_b,$$

where the total number P_T of the *tetR* promoter in the downstream module is given by the sum of the numbers of the free and bound promoters: $P_f + P_b$. The transcription and translation processes for each repressor gene are assumed to be identical. The parameter values used for simulations are listed in Table S1. The parameters were modified to increase the retroactivity by reducing the expression levels of the repressor molecules (a_{tr} : 29.97 → 2.97, K_M : 40 → 10, eff : 20 → 10).

S2 Linear relationship between C' and P_T

This section takes into account the *intermediate* reaction steps of oligomerization and enzyme-mediated degradation.

*kkim@u.washington.edu

†hsauro@u.washington.edu

a_{0tr}	0.03 min^{-1}	Basal transcription rate
a_{tr}	$3 - 0.03 = 2.97$ min^{-1}	Maximum transcription rate is set to 3 min^{-1}.
K_M	10	Number of repressor molecules corresponding to the half maximal transcription
kd_{mRNA}	$\frac{\log(2)}{\tau_{mRNA}}$ min^{-1}	Degradation rate constant of mRNA
τ_{mRNA}	2 min	Half life time of mRNA
k_{tl}	$eff\frac{\log(2)}{\tau_{mRNA}}$ min^{-1}	Translation rate per mRNA
eff	10	Translation efficiency: Number of repressor molecules translated per mRNA during mRNA life time
kd_{prot}	$\frac{\log(2)}{\tau_{prot}}$ min^{-1}	Degradation rate constant of repressor molecules
τ_{prot}	10 min	Repressor molecule half life time
k_{on}	0.166 min^{-1}	Promoter-repressor binding constant [2]
k_{off}	1.66 min^{-1}	Promoter-repressor unbinding constant for a given dissociation constant $K_d = k_{off}/k_{on} = 10$
P_T	0 or 100	Number of *tetR* promoters in a downstream module

Table S1. Parameters of the repressilator BioModel

S2.1 Oligomer TF

We consider a dimer TF (the results obtained below can be generalized to any other oligomer types). The corresponding model can be expressed as:

$$\xrightarrow{\alpha} X \xrightarrow{\gamma X} \emptyset$$
$$X + X \underset{k_2 X_2}{\overset{k_1 X^2}{\rightleftharpoons}} X_2 \xrightarrow{\gamma_2 X_2} \emptyset$$
$$X_2 + P_f \overset{K_d}{\rightleftharpoons} P_b,$$

where the reactions from the top represent the monomer translation and degradation, dimerization, dimer degradation, and promoter-binding-unbinding reactions. K_d denotes the dissociation constant. The time evolution of the total transcription factor (monomer units) ($Y = X + 2X_2 + 2P_b$) is governed by

$$\frac{dY}{dt} = \alpha - \gamma X - 2\gamma_2 X_2.$$

The response time constant of Y becomes

$$\tau_{P_T} = -\left[\frac{d(\alpha - \gamma X - 2\gamma_2 X_2}{dY}\right]^{-1} = \left[\gamma\frac{dX}{dY} + 2\gamma_2\frac{dX_2}{dY}\right]^{-1}. \tag{S1}$$

Under the assumption of the equilibrium of X_2 and P_b, we obtain

$$X_2 = \frac{k_1}{k_2 + \gamma_2}X^2, \quad \text{and} \quad P_b = \frac{P_T X_2}{K_d + X_2} \equiv P_T f(X). \tag{S2}$$

By substituting the first equation in the above to Eq. (S1), the time constant is obtained as

$$\tau_{P_T} = \left[\gamma + \frac{4\gamma_2 k_1 X}{k_2 + \gamma_2}\right]^{-1}\frac{dY}{dX},$$

which becomes, by using $Y = X + 2X_2 + 2P_b$ and Eq. (S2),

$$\tau_{P_T} = \tau_0\left[1 + \frac{2f'(X)}{1 + \frac{4k_1 X}{k_2 + \gamma_2}}P_T\right], \tag{S3}$$

where τ_0 denotes the time constant in the case without any promoter:

$$\tau_0 = \frac{1 + \frac{4k_1 X}{k_2+\gamma_2}}{\gamma + \frac{4\gamma_2 k_1 X}{k_2+\gamma_2}}.$$

Here the steady state concentration of X is independent of P_T. Equation (S3) shows that the extra capacitance C' is proportional to P_T (by noting that $\tau_{P_T} = RC_T = R(C + C')$ and $\tau_0 = RC$).

S2.2 Oligomer TF under an inhibitory self-regulation

The above case is extended by applying an inhibitory self-regulation. The translation rate α is modified to

$$\alpha \to v(X_2) = \frac{\alpha}{1 + \beta X_2},$$

where β is introduced to turn on and off the regulation. By following the identical procedure in the previous section, the time constant becomes

$$\tau_{P_T} = \tau_0 \left[1 + \frac{2f'(X)}{1 + \frac{4k_1 X}{k_2+\gamma_2}} P_T \right],$$

where τ_0 becomes modified to

$$\tau_0 = \frac{1 + \frac{4k_1 X}{k_2+\gamma_2}}{-\frac{dv(X_2)}{dX} + \gamma + \frac{4\gamma_2 k_1 X}{k_2+\gamma_2}}.$$

Here the steady state concentration of X is independent of P_T. The extra capacitance C' is proportional to P_T (by noting that $\tau_{P_T} = RC_T = R(C + C')$ and $\tau_0 = RC$).

S2.3 Degradation-tagged TF

We consider a monomer TF under directed degradation by proteases. The corresponding model can be expressed as:

$$\xrightarrow{\alpha} X$$

$$X + E \underset{k_2[XE]}{\overset{k_1 X \cdot E}{\rightleftharpoons}} XE \xrightarrow{\gamma[XE]} E$$

$$X + P_f \overset{K_d}{\rightleftharpoons} P_b,$$

with the total number of the proteases $E_T (= E + [XE])$ fixed. The time evolution of the total TF ($Y = X + [XE] + P_b$) is governed by

$$\frac{dY}{dt} = \alpha - \gamma[XE].$$

The response time constant of Y becomes

$$\tau_{P_T} = -\left[\frac{d(\alpha - \gamma[XE])}{dY} \right]^{-1} = \left[\gamma \frac{d[XE]}{dY} \right]^{-1}.$$

Under the assumption of the equilibrium of $[XE]$ and P_b, $[XE]$ and P_b can be expressed in terms of X as like Eq. (S2). By following the same way as above, the time constant is shown to increase by the amount proportional to P_T as like Eq. (S3).

S2.4 Multiple operators

Consider a dimer TF that can bind two operator sites with different affinities. The binding-unbinding process between the TF and the promoter can be modeled as

$$X + P_0 \rightleftharpoons P_1$$

$$X + P_0 \rightleftharpoons P_2$$

$$P_1 \rightleftharpoons P_{12}$$

$$P_2 \rightleftharpoons P_{12},$$

where P_0, P_i, and P_{12} denote promoters that are free, occupied at the i-th operator site, and fully occupied, respectively. The equilibrium constant of each reaction from top to bottom is denoted by K_1, K_2, K_3, and K_4. By using that the total promoter concentration P_T equals $P_0 + P_1 + P_2 + P_{12}$, we obtain

$$P_b \equiv P_1 + P_2 + P_{12} = \frac{P_T X}{X + K_d} \equiv P_T f(X), \tag{S4}$$

where

$$K_d \equiv [K_1 + K_2 + K_1/K_3]^{-1}.$$

Here K_1/K_3 is equal to K_2/K_4. The response time constant τ_{P_T}, given by

$$\tau_{P_T} = -\left[\frac{d(\alpha - \gamma X)}{dY}\right]^{-1} = \tau_0 \frac{dY}{dX},$$

is re-written as

$$\tau_{P_T} = \tau_0 (1 + f'(X) P_T). \tag{S5}$$

by using $Y = X + P_b$. If there were n-operators and the TF is an oligomer, the same derivation can be applied resulting in Eq. (S5).

References

[1] Le Novère N, Bornstein B, Broicher A, Courtot M, Donizelli M, et al. (2006) BioModels Database: a free, centralized database of curated, published, quantitative kinetic models of biochemical and cellular systems. Nucleic Acids Res 34: D689–D691.

[2] Elf J, Li GW, Xie XS (2007) Probing transcription factor dynamics at the single-molecule level in a living cell. Science 316: 1191–1194.
